# Post-diagnostic support for dementia in the UK: mapping access, delivery and gaps in provision

**DOI:** 10.1186/s12913-026-15331-2

**Published:** 2026-08-08

**Authors:** Melissa Melville, Dominika Valacsay, Josefine Funder Nissen, Nahid Zokaei, Lily Mainstone-Cotton, Aimee Spector

**Affiliations:** 1 Department of Clinical Health Psychology, University College London, London, UK; 2 Research and Development Department, North East London NHS Foundation Trust Goodmayes Hospital, Ilford, UK; 3 Division of Psychiatry, University College London, London, UK; 4 Brain+ A/S, Svanevej 22, 2, Floor DK-2400, Copenhagen, NW Denmark

**Keywords:** Post-diagnostic support, Dementia, Cognitive stimulation therapy, Service provision, Implementation barriers, Non-pharmacological intervention, NHS services, Workforce capacity

## Abstract

**Background:**

Dementia is a progressive condition affecting approximately 982,000 people in the UK, with substantial implications for people living with dementia, carers, and healthcare systems. Effective post-diagnostic support is essential for managing symptoms and maintaining quality of life, yet services remain inconsistent, fragmented, and highly variable across regions, particularly in rural and underserved areas. Cognitive Stimulation Therapy (CST) is the only non-pharmacological intervention recommended by the National Institute for Health and Care Excellence (NICE) to improve cognition, independence and wellbeing in people with dementia. However, a 2020 survey indicated substantial variation in its availability and delivery.

**Objectives:**

To investigate the availability, delivery, and challenges of post-diagnostic support for dementia in the UK, with a particular emphasis on CST.

**Methods:**

An online survey with a mix of open and closed questions was conducted between August and December 2024 among healthcare professionals working in NHS Trusts, memory clinics, care-homes, non-profit organisations and dementia services across the UK. The questionnaire collected information on post-diagnostic dementia support, including the availability and delivery of CST, psychological support, staffing, service integration and perceived barriers to service provision. Quantitative data were summarised descriptively, and free-text responses were analysed using descriptive content analysis.

**Results:**

129 responses were received from staff across 71 distinct services across the UK, including 45 of the 202 NHS Trusts and third-sector dementia organisations (e.g. the Alzheimer’s Society). All available data, including partial responses, were included in the analysis. Respondents reported variation in the availability of post-diagnostic support across UK dementia services. 65.5% (55/84) reported that their organisation delivered CST. Of those, 58.5% (24/41) reported running only a single CST group programme at a time. Reported waiting times for post-diagnostic support varied widely, with a mean of 13.7 weeks and a range from 2 to 100 weeks. Overall, the findings highlight reported delays in access to post-diagnostic support, variation in the availability of CST, and persistent challenges related to staffing capacity and funding constraints.

**Conclusion:**

These findings suggest substantial variation in the reported availability and delivery of post-diagnostic support across UK dementia services. There is an urgent need for increased funding, workforce expansion, and clearer national standards for post-diagnostic dementia care to ensure that people living with dementia can access timely, evidence-based support that helps them maintain independence, manage symptoms, and sustain quality of life following diagnosis.

**Supplementary Information:**

The online version contains supplementary material available at https://doi.org/10.1186/s12913-026-15331-2.

## Introduction

### Background

Dementia affects approximately 982,000 people in the UK, placing considerable strain on individuals, carers, and the healthcare system [1]. Receiving a dementia diagnosis can also have substantial psychological and social consequences, including depression, anxiety, and uncertainty about future health, independence, and care needs [2–5]. Given these challenges, timely and effective post-diagnostic support is widely recognised as a critical component of dementia care, ideally beginning soon after diagnosis and continuing flexibly throughout the course of the condition to help individuals and their families maintain wellbeing and quality of life [3–5].The 2022 World Alzheimer Report defines post-diagnostic support as an umbrella term encompassing formal and informal services and information designed to promote the health, social, and psychological wellbeing of people with dementia and their carers following diagnosis [6]. Post-diagnostic support may include information and psychoeducation; cognitive and psychosocial interventions; medication and symptom management; community and social support; care coordination; legal and financial planning; carer support and respite; and advance care planning [7].

International and national policy frameworks increasingly emphasise the importance of strengthening post-diagnostic support. The 2022 World Alzheimer Report [6] called for governments to build robust models, using the key action areas of the WHO Global Dementia Action Plan as a framework [8]. In England, national initiatives including the Prime Minister’s Challenge on Dementia and NHS England’s dementia priorities have similarly highlighted the need for accessible, person-centred post-diagnostic support [9].

Despite these policy ambitions, many individuals report feeling unsupported following a dementia diagnosis, with limited guidance to help them navigate available services and long waiting times for support [10]. Access to high-quality post-diagnostic support remains inconsistent across regions and services, with rural and socioeconomically deprived areas facing particular barriers to access [9, 11]. For example, in Scotland, all people diagnosed with dementia are entitled to a minimum of one year of post-diagnostic support, a provision that is not available elsewhere in the UK [12]. Differences in service design and commissioning may contribute to these inequalities. Some memory services are primarily commissioned to provide diagnostic assessment rather than ongoing support, leaving them without the workforce, budget, or infrastructure required to deliver post-diagnostic support [13].

Within the spectrum of post-diagnostic support, structured psychosocial and rehabilitation-based approaches play an important role in helping people maintain function, independence, and wellbeing. Recent guidance from the WHO and the World Alzheimer Report emphasises dementia rehabilitation as an important but often overlooked component of post-diagnostic support, highlighting multidisciplinary, goal-oriented approaches that support everyday functioning and independence following diagnosis [14, 15]. NICE guidelines similarly recommend considering cognitive rehabilitation or occupational therapy to support functional ability in people with mild to moderate dementia [7].

CST is currently the only non-pharmacological intervention recommended by NICE guidelines to support cognition, independence and well-being in people living with mild to moderate dementia [7]. CST is a structured, evidence-based group intervention involving themed activities designed to stimulate thinking, concentration, and memory [16]. CST now has a substantial international evidence base demonstrating benefits for cognitive functioning and quality of life, alongside evidence of cost-effectiveness ([17–18]). Despite this evidence and policy recommendation, implementation of CST remains inconsistent across the UK.

A recent Memory Services Spotlight Audit [19] found that although 62% of services reported delivering CST, only 31% of people living with dementia were offered the intervention. The audit also found that post-diagnostic monitoring is available in 53% of services; however, in 32% of these services monitoring is restricted to individuals prescribed dementia-specific medication.

Taken together, these findings suggest a substantial gap between policy ambition and the reality of post-diagnostic support provision. Understanding how services currently deliver post-diagnostic support, and how evidence-based interventions such as CST are implemented in practice, is essential to inform future service development.

### Aims and objectives

This study aimed to explore the current landscape of post-diagnostic support for people with dementia across the UK, with a particular focus on CST. Specifically, it sought to:

1. Examine how post-diagnostic support is currently delivered and identify key barriers and considerations.
2. Understand how CST is implemented in practice and the factors influencing its provision.

## Methods

### Ethics

This survey was conducted as an amendment to a wider programme of research evaluating Compassion Focused Therapy for mood difficulties in people living with dementia. The survey was undertaken to better understand the availability of post-diagnostic support and CST across UK dementia services, providing context for the wider programme of research. Ethical approval for the larger study, including this amendment, was granted by the Health Research Authority Research Ethics Committee (London Riverside, 23/LO/0535). Participation in the survey was voluntary, and informed consent was implied by participants through the completion and submission of the survey.

### Questionnaire design

The questionnaire consisted of both closed and open-ended questions designed to generate quantitative data and free-text responses. It was developed specifically for this project through a collaboration between the research teams at UCL and Brain+, informed by their academic and clinical expertise in dementia care and CST and drawing on prior surveys, national audits and service standards for memory services [7, 10, 19]. The questionnaire was refined through iterative review to ensure clarity and relevance. Draft questions were reviewed informally by clinicians from local memory services, whose feedback informed revisions to question wording, response options and questionnaire structure, and helped streamline completion time to under 10 min. This process was intended to establish the face and content validity of the questionnaire. Formal psychometric validation (e.g., assessment of construct validity or reliability) was not undertaken.

The questionnaire included closed questions on availability, organisation and delivery of post-diagnostic support, including CST, psychological support, waiting times, staffing, workforce composition, community integration and funding. Open-ended questions invited respondents to describe additional post-diagnostic services, barriers and facilitators to delivering post-diagnostic support and CST, approaches used to address these challenges, and any other comments regarding service provision. The full questionnaire is provided as Supplementary File 2.

**Fig. 1:**
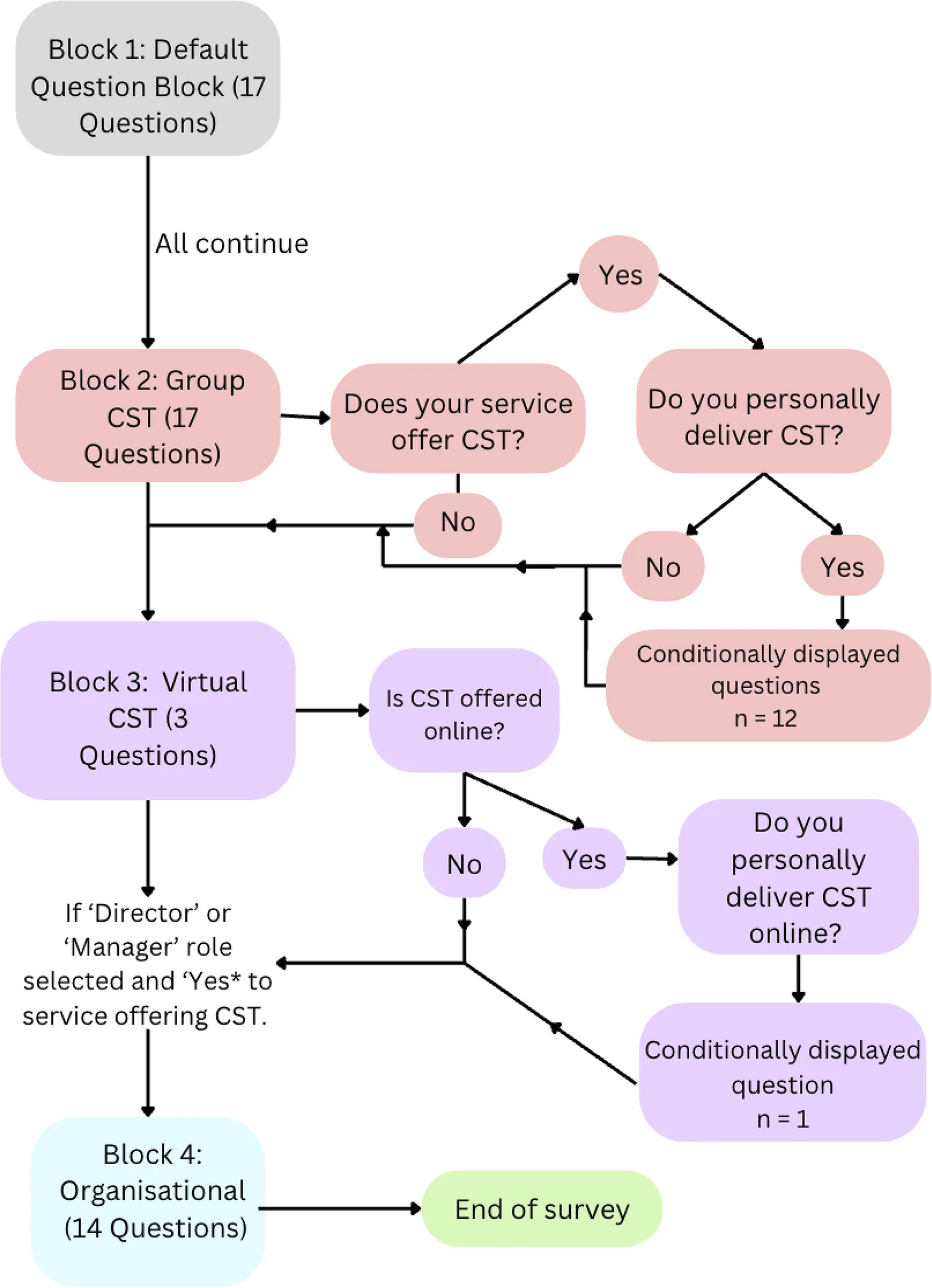
Survey logic and conditional branching

As shown in Fig. 1, the questionnaire was organised into four blocks: (1) respondent characteristics and post-diagnostic support provision; (2) CST availability, delivery, quality and implementation; (3) virtual CST; and (4) organisational questions for respondents with managerial or director-level responsibilities. Respondents progressed through these blocks using conditional branching, whereby some sections were displayed only to respondents whose role or service context was relevant.

### Recruitment and participants

The survey was hosted on Qualtrics and remained open between August and December 2024. In the UK, key elements of dementia care, including diagnostic assessment, initiation and monitoring of medication and time-limited post-diagnostic support, are commonly delivered through NHS Trusts, typically via memory services within mental health or community trusts. NHS trusts are regional organisations responsible for providing health services within defined geographical areas. These services operate alongside a wider network of social care, community services, and voluntary sector organisations that contribute to dementia care and support. To ensure wide coverage across these Trusts, the survey was distributed via national NHS mailing lists and bulletins, including the Mental Health Network NHS Confederation Bulletin and the NHS Healthcare Leaders Bulletin, which reach a wide range of dementia professionals across the UK, including clinicians, psychologists, dementia nurses, occupational therapists, service managers, and commissioners. Additional dissemination occurred through the British Psychological Society’s Faculty of Psychiatry of Older People, whose membership spans all UK nations and includes regional groups that circulate communications to local clinical networks. Where possible, we also contacted the primary contact listed for each memory clinic or service across all NHS Trusts, requesting that they circulate the survey to relevant staff involved in post-diagnostic support. The invitation email highlighted the study’s relevance to post-diagnostic support and CST, which was intended to appeal to staff actively engaged in these services. Allied health assistants, care support workers, and general practitioners were not specifically targeted unless they were involved in delivering or coordinating post-diagnostic support. The survey did not apply strict inclusion or exclusion criteria, reflecting a pragmatic approach to capture perspectives from any staff involved in post-diagnostic support. Due to the method of distribution, it was not possible to determine the total number of staff or services reached; therefore, a formal response rate could not be calculated. Respondents were healthcare professionals involved in the delivery or coordination of post-diagnostic support, including CST. All available data, including partial completions, were analysed to ensure comprehensive coverage of respondents’ perspectives.

Respondents were asked to report their role within the organisation, selecting from the following categories: therapist, nurse, manager, director, or other. These categories were intended to broadly capture respondents’ professional function within dementia services rather than provide an exhaustive list of professional titles, recognising that some individuals may hold both clinical and managerial responsibilities. Free-text responses provided under the “Other” category were reviewed and, where appropriate, reclassified to align with existing categories. Specifically, respondents who indicated their role as Clinical Psychologists or Occupational Therapists were reclassified as therapists to reflect their shared involvement in delivering psychological or rehabilitative interventions within post-diagnostic dementia care. Roles that did not clearly align with the predefined categories were retained within the “Other” category.

### Data analysis

All responses were exported to Microsoft Excel for data cleaning and removal of duplicate entries. Duplicate responses were assessed through manual review of patterns of identical responses across survey items and associated timestamps. Quantitative data were summarised descriptively. As the survey incorporated conditional branching, not all respondents were presented with every question; consequently, denominators vary across analyses and are reported alongside the relevant results.

Open-ended responses were analysed using inductive descriptive content analysis. Given that the data comprised brief free-text responses, the analysis was intended to provide a descriptive summary of recurring categories. Two members of the research team independently reviewed and coded all free-text responses, with codes generated inductively from the data rather than using a pre-existing coding framework. The reviewers then compared and discussed their coding, resolving any differences through consensus. Similar codes were grouped into broader descriptive categories relating to barriers, facilitators, and service delivery experiences. These categories informed the descriptive findings presented in the Results.

## Results

### Participants

A total of 129 responses were received. Of these, 78 provided complete data, answering all questions relevant to their role and service based on the survey’s branching logic. The remaining 51 responses were partial. Most partial responses completed Blocks 1 and 2, with respondent numbers decreasing in later sections. The reduction in respondent numbers across later sections reflected both survey discontinuation and the use of conditional branching.

Responses were received from 71 distinct services across the UK, including 45 of the 202 NHS Trusts with representation from both urban centres and rural areas such as Cornwall, Norfolk, Powys, and Guernsey. Most organisations contributed one or two responses, with the exception of North-East London NHS Foundation Trust, which accounted for 7.2% of the total sample (*n* = 9/125). Of the 125 respondents who reported their organisation, the majority were based in England (93.6%, 117/125), with smaller contributions from Scotland (4.8%, 6/125) and Wales (0.8%, 1/125). One response was received from Guernsey (0.8%, 1/125), while no responses were recorded from Northern Ireland. As some organisations contributed more than one respondent, all analyses are presented at respondent level unless otherwise stated.

The most commonly reported staff roles were Therapist (39.5%, 51/129) and other (21.7%, 28/129), followed by Nurse (20.9%, 27/129) and Manager (16.3%, 21/129). Only two respondents identified as Directors (1.6%, 2/129). Respondents in the “Other” category included Clinical Leads, Assistant Psychologists, Dementia Navigators, and Information and Education Advisors.

### Availability of post-diagnostic support

When asked which types of post-diagnostic support were available within their service (respondents could select multiple responses), the most commonly selected form of support was dementia information and signposting (67.6%, *n* = 69/102). Cognitive interventions, such as life story work, CST, reminiscence, and orientation-based approaches aimed at supporting cognitive engagement, were selected by 56.9% (*n* = 58/102) of respondents. Half of respondents (50%, *n* = 51/102) selected dementia-specific psychological support, including individual or group counselling and therapeutic activities such as music or art therapy. Dementia-specific physical health services were selected by 22.5% (23/102) of respondents and included interventions focused on physical functioning and wellbeing, including physiotherapy, occupational therapy or support addressing mobility and functional independence. 35 open-ended responses described additional forms of post-diagnostic support, including care coordination, dementia day-care centres, admiral nursing, assistive technology, occupational health, physiotherapy, Acceptance and Commitment Therapy (ACT), and mindfulness-based workshops.

### Integration with community services

Respondents were asked whether their service was integrated with local dementia charities or community groups. Of the 84 respondents who answered this question, 66.7% (56/84) reported some form of collaboration with voluntary or community organisations, while 33.3% (28/84) reported no such collaboration. In this context, “integration” refers to respondents’ reports of collaborative working or links with community organisations, rather than formal service-level integration.

Among the 56 respondents reporting collaboration with community services, the Alzheimer’s Society was the most frequently mentioned partner organisation (32.1%, 18/56), followed by Carers UK (30.4%, 17/56) and Age UK (16.1%, 9/56). Respondents could report more than one partner organisation.

Open-ended responses described collaboration with community organisations in several ways, including signposting to dementia cafés, social groups, carer support and exercise programmes, alongside more formal partnerships such as commissioned post-diagnostic support, embedded dementia navigator roles and joint NHS-community initiatives.

### Respondents’ perceptions of post-diagnostic support

Of the 121 respondents who answered the question on satisfaction with post-diagnostic support, 52.9% (64/121) reported dissatisfaction with the support provided by their service. Of these respondents, 75% (48/64) provided additional comments via a free-text question. The most frequently reported barrier from free-text responses was limited access to post-diagnostic support (79.2%,38/48), commonly attributed to commissioning constraints. Several respondents reported that their services were commissioned primarily for diagnosis and therefore relied on referral or signposting to third-sector organisations for post-diagnostic support: “*We are commissioned as a diagnostic-only service*,* so the support I offer is very limited*” (Therapist 2); “*We are only contracted to diagnose patients and signpost to other services*” (Manager 1) and “*It’s outsourced to the Alzheimer’s Society*” (Therapist 5). Another respondent commented, *“Support is offered by third-sector partners*,* but there is a lot of room for improvement”* (Therapist 12). Respondents also reported limited availability of evidence-based interventions, including CST and psychological support: “*We currently have no CST provision and no psychological offering*” (Therapist 4), while another noted that “*Unless referred into Community Mental Health Team in crisis*,* no post-diagnostic support is available*” (Therapist 19).

Respondents described limited resources, increasing referral volumes and restricted capacity for post-diagnostic support, which could affect the extent of follow-up offered after diagnosis. One respondent stated that “*Huge pressures on the waiting list for diagnosis mean there is little left for post-diagnostic pathways*” (Therapist 12), while another explained that “*The volume of referrals means that resource is focused on diagnostic services and there is very limited capacity for post-diagnostic support*” (Clinical Lead 3). Several respondents reported staffing constraints including limited multidisciplinary input: “*Very few roles other than nursing and medics work from a medical model*” (Clinical Lead 1). A small number of respondents described initiatives to address these challenges, including staff training and role development: “*I have been in post four months with the remit of upskilling existing staff and bidding for new roles*” (Therapist 4).

Respondents described challenges in delivering and monitoring post-diagnostic support despite reporting the availability of interventions such as CST. One respondent commented, “*We offer several groups including CST*,* but due to pressures also signpost to the third sector and are unable to monitor the uptake. I fear people are missing out*” (Therapist 11).

Equity of access was raised by respondents. One noted that *“Significant diversity in population means English speakers are offered CST more than other groups”* (Nurse 1). Respondents described the impact of limited post-diagnostic support for people living with dementia and their carers: *“Post- diagnostic support is non-existent*,* and many of our clients have highlighted this and the distress it causes” (Therapist 7).*

### Support for anxiety and depression

Respondents were asked specifically whether their service provided psychological support for people with dementia experiencing symptoms of anxiety or depression, 78.7% (*n* = 70/89) of respondents reported that such support was available, while 21.3% (*n* = 19/89) reported that it was not. Among respondents reporting the availability of psychological support, the most commonly reported interventions were individual or group counselling or psychotherapy (60.6%, *n* = 40/66) followed by behavioural interventions (50%, *n* = 33/66). Life story work was also reported by 40.9% (*n* = 27/66) while smaller numbers of respondents reported animal-assisted therapy (9.1%, *n* = 6/66) and music or art therapy (6.1%, *n* = 4/66).

### Waiting times for post-diagnostic support

Among respondents, 65.2% (*n* = 60/92) reported waiting times for accessing post-diagnostic support, while 34.8% (*n* = 32/92) reported that post-diagnostic support was immediately available within their service. Among respondents reporting waiting times, delays ranged from 2 to 100 weeks. 37.8% (14/37) reported waiting times between 6 and 12 weeks. The mean reported waiting time was 13.7 weeks.

### Clinical psychologists available within NHS services

Among respondents, 32.9% (*n* = 27/82) reported that no clinical psychologists were available within their service. A further 25.6% (*n* = 21/82) reported that their service had one clinical psychologist, while 41.5% (*n* = 34/82) reported having two or more psychologists within their service.

### Availability and delivery of CST

Overall, 65.5% (*n* = 55/84) of respondents reported that their organisation delivered CST. Among respondents who provided further information on CST delivery, 70.7% (*n* = 29/41) reported delivering CST frequently (defined as at least once per week), 24.4% (*n* = 10/41) reported delivering CST occasionally (less than once per week), and 4.9% (*n* = 2/41) reported that CST had not yet been delivered. Among respondents whose organisations delivered CST, 58.5% (*n* = 24/41) reported running a single CST group at a time.

### Virtual (online) CST

Most respondents (91.25%, *n* = 73/80) reported that their organisation did not offer virtual CST. Among respondents whose organisations delivered virtual CST (*n* = 7), three reported being directly involved in its delivery. One respondent reported delivering one group annually, while the remaining two reported that virtual CST had been used primarily during the COVID-19 pandemic, with little subsequent demand.

### Current CST funding

Information about CST funding was provided through free-text responses from 15 respondents. Of these, (33.3%, *n* = 5/15) described funding through local commissioning or service-level contracts. Among respondents who commented on future funding (*n* = 9), none anticipated changes in CST funding for the following year. Responses reported that CST funding was provided through NHS or Trust-level budgets, local commissioning arrangements, and external organisations such as Age UK. Several respondents reported that no dedicated funding streams for CST were available.

### Challenges to implementing CST

Respondents identified several barriers to CST delivery. The most commonly reported were high referral volumes (83.8%, 31/37), the need to provide other forms of support alongside CST (51.4%, 19/37), insufficient staffing or limited staff time (27.0%, 10/37), limited access to suitable venues (18.9%, 7/37), transport difficulties for attending sessions (18.9%, 7/37), broader resource constraints (13.5%, 5/37), participant attendance and engagement (13.5%, 5/37), competing clinical demands (5.4%, 2/37), and lack of management prioritisation (5.4%, 2/37). Some respondents also reported difficulties delivering CST online.

### Strategies to overcome challenges with CST delivery

Strategies for addressing challenges to CST delivery were described in free-text responses. Reported strategies included recruiting or reallocating staff, tailoring session resources to needs and capacity, adapting session structures, improving referral pathways, engaging carers and people with dementia, and securing additional funding. Some respondents also described practical approaches to addressing logistical barriers. No respondents reported plans to expand CST provision within their organisation, although two respondents described developing culturally tailored or bespoke CST sessions and increasing staff involvement through third-party partnerships.

**Fig. 2:**
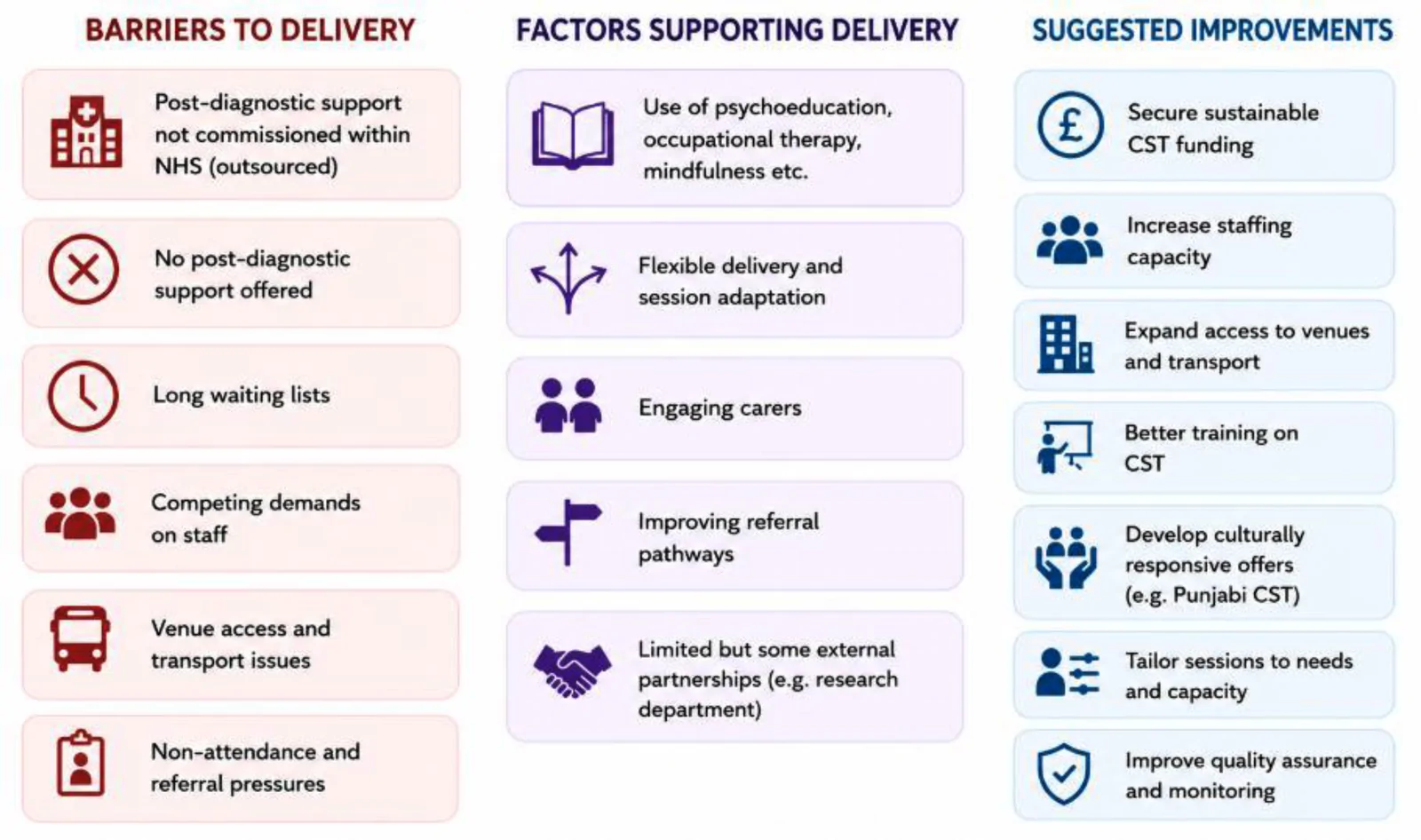
Descriptive summary of recurring categories identified from free-text responses relating to barriers to post-diagnostic support and CST delivery, factors supporting delivery, and suggested improvements

Figure 2 summarises the recurring categories identified from free-text responses relating to barriers to delivery of post-diagnostic support, factors supporting delivery, and suggested improvements.

## Discussion

This study aimed to evaluate the current state of post-diagnostic support for people with dementia across the UK, with a particular focus on the availability, delivery, and challenges of CST. Respondents reported that services offered a range of post-diagnostic support, including psychoeducation, psychosocial interventions, and care coordination. Consistent with prior research [10], half of the respondents expressed dissatisfaction with the current provision in their service. In free-text responses, many cited limited-service availability.

In the UK, national guidelines emphasise the importance of post-diagnostic care for people with dementia [7] Despite this, respondents frequently reported that their services were commissioned primarily to provide diagnostic assessment, with no mandate or funding to deliver ongoing follow-up care. Nearly one in five respondents (18.8%) reported that no formal post-diagnostic support was available within their service, whereas 37.5% reported that support was not delivered within the service but outsourced to external providers. Where support was outsourced, respondents noted that coordination between services and external providers was often limited, with few mechanisms in place to monitor uptake. While this study did not measure the number of people receiving post-diagnostic care, these findings suggest variation in access to post-diagnostic support across UK dementia services. This is consistent with previous reports showing that 61% of people with dementia reported not receiving sufficient support [20]. Inconsistent commissioning and fragmented service arrangements may therefore limit access to interventions designed to help people manage symptoms, maintain independence, and live as well as possible following diagnosis. In turn, gaps in post-diagnostic support may reduce quality of life, limit independance, and increase pressure on families and caregivers. These findings highlight the disconnect between policy ambitions for accessible person-centred post-diagnostic support and the reality of service provision, reinforcing the need for clearer national standards and coordinated commissioning of post-diagnostic dementia care. Among respondents, dementia information and signposting was the most commonly reported form of post-diagnostic support available within their service. However, the absence of even this basic level of support in a substantial proportion of services reflects wider evidence that many people experience a “void” following diagnosis, unsure how to navigate available services or care pathways [21]. Qualitative research suggests that written information alone is often insufficient following a dementia diagnosis, with people with dementia and their carers emphasising the need for ongoing guidance and personalised support to interpret information and access appropriate services [22]. Studies have also highlighted that carers may frequently struggle to navigate various sources of information and that professionals may rely heavily on signposting to written or online materials, despite carers’ preference to receive information through direct engagement with professionals [23].Without timely information about available support and care pathways, individuals may struggle to engage with services designed to help them manage symptoms, maintain independence, and plan for future care.

CST remains the only NICE recommended non-pharmacological intervention for people with dementia [7, 16] with accreditation standards for UK memory services (Memory Services National Accreditation Programme, MSNAP) linked to provision of CST [24]. Our findings indicate that 65.5% of respondents reported that their organisation delivers CST. These figures exceed those reported in the recent national audit [19], which found that just 31% of people living with dementia were offered CST despite its endorsement in NICE guidelines [7]. Respondents identified staffing shortages, logistical challenges, and inadequate funding as the primary barriers to broader implementation, reflecting concerns previously noted by Holden et al. [25].

The wide variation in waiting times for access to CST and broader post-diagnostic support is concerning, with one respondent reporting a delay of up to 100 weeks. This echoes findings from Frost et al. [10], who reported that access targets for dementia participants were rarely met. Given that CST has demonstrated benefits for cognition and quality of life in people living with mild to moderate dementia [17], substantial delays in delivery may reduce the opportunity for people to benefit when they are at a stage most responsive to the intervention.

Barriers to CST implementation reported by respondents, including staffing shortages, lack of funding, and long waits, were also identified in a recent practitioner-focused study by Kelly et al. [26], which found that while 95% of CST-trained clinicians intended to deliver CST, fewer than 50% actually did. Our findings extend this evidence by suggesting that structural and operational barriers may prevent CST from being delivered consistently in line with the manual’s recommendations [27]. For instance, while CST is designed as a 14-session programme delivered twice weekly, many respondents reported that their service delivered sessions only once per week, suggesting deviation from the standard protocol that may reduce the intervention’s intended intensity and potentially reduce its effectiveness. Free-text responses commonly described staffing shortages, logistical challenges and inadequate funding as barriers to implementation.

Virtual delivery of CST has been explored as an alternative to face-to-face groups. Fisher et al. [28] demonstrated the feasibility and acceptability of culturally adapted virtual CST in India and Brazil, although at the time of writing it had largely been implemented in response to the COVID-19 pandemic. While many participants and caregivers found virtual CST acceptable during periods of social isolation, some caregiver dyads and facilitators expressed a preference for in-person sessions.

Virtual CST may nevertheless help address some of the barriers identified in this study, particularly transport difficulties, geographical distance, and limited access to suitable venues. However, challenges such as digital exclusion, limited access to technology, and lower digital literacy among some older adults may restrict participation. Despite early interest in hybrid delivery models following the pandemic [29], our findings suggest that virtual CST has not yet been widely adopted in routine practice.

Although half of respondents selected dementia-specific psychological therapies as part of their services post-diagnostic offer, this figure should be interpreted cautiously. A substantial proportion of survey respondents were therapists, which may bias estimates towards services with stronger psychosocial offerings. Existing UK research indicates substantial regional variation in access to psychological support [19, 21], suggesting that provision may be far less consistent than our sample implies. Workforce constraints likely play an important role. Many respondents reported limited availability of psychologists within their services, which aligns with national audits and workforce reviews documenting longstanding shortages of dementia-trained psychological staff across UK services [19, 21]. The limited availability of specialised staff may restrict access to timely psychological interventions and contribute to delays in receiving post-diagnostic support [25]. Although psychological support is an important component in managing symptoms such as anxiety, depression, and behavioural challenges, its provision remains constrained. Addressing this gap through strategic workforce planning and investment is essential to improve both the quality and equity of dementia care.

Physical health services were reported by relatively few respondents, highlighting potential gaps in mobility support, falls prevention, and management of comorbidities, which are central to holistic post-diagnostic care. These findings suggest that elements of dementia rehabilitation, as described in the WHO and World Alzheimer reports [6, 8], are not consistently implemented in UK memory services. Dementia rehabilitation typically involves multidisciplinary approaches that support cognitive, physical, and functional abilities, with the aim of helping people maintain independence and continue everyday activities following diagnosis. Our findings suggest that physical and functional rehabilitation elements are not consistently available within post-diagnostic services. Addressing this gap will require clearer commissioning structures, better integration of physical, cognitive, and psychological care, and investment in multidisciplinary workforce capacity. Embedding rehabilitation within post-diagnostic pathways may help people living with dementia maintain function, independence, and quality of life post diagnosis.

### Strengths and limitations

This study has several strengths, including its national scope and the use of both closed and open-ended questions, enabling exploration of both the breadth of post-diagnostic support provision and the challenges encountered by practitioners. There were responses from memory clinics across NHS trusts and organisations in the UK, including rural and less populated areas such as Cornwall, Norfolk, Powys, and Guernsey. Most organisations contributed one to three responses, reducing the influence of any single trust on the overall findings.

The survey was distributed via national NHS mailing lists and professional networks rather than through a formal sampling frame, representing an opportunistic approach to recruitment. This strategy enabled broad dissemination and captured perspectives from a range of dementia professionals across the UK. However, this recruitment approach also has several limitations. Given that the total number of services or staff who received the survey invitation is unknown, a formal response rate could not be calculated. In addition, geographical representation was uneven, with some areas, including Northern Ireland and Wales, under-represented. Respondents may also have been more engaged with dementia services or more motivated to participate than non-responders, introducing the possibility of selection bias.

All available data, including partial responses, were included to maximise the breadth of respondents’ perspectives. Most partial responses completed the initial sections of the questionnaire, with respondent numbers decreasing in later sections because of both survey discontinuation and conditional branching. Although completed responses were retained for analysis, missing data may have introduced non-response bias if respondents who did not fully complete the questionnaire differed systematically from those who did. This may have reduced the representativeness of the sample and should be considered when interpreting the findings. In addition, the questionnaire was developed specifically for this study and underwent clinician review to establish face and content validity. However, no formal psychometric validation, including assessment of construct validity and reliability, was undertaken. The questionnaire collected the number of staff rather than full-time equivalents; consequently, reported numbers include part-time staff and those fulfilling multiple roles, meaning that actual staffing capacity may be lower than the headcount suggests.

Given the relatively low reported provision of post-diagnostic support among participating organisations, it is possible that non-responding services differed in the availability of support offered. As respondents may have had a greater interest in dementia services than non-responders, the findings may overestimate the availability of post-diagnostic services nationally. The questionnaire did not include structured questions on carer-specific support or respite services, limiting our ability to capture the full scope of post-diagnostic support available to carers.

The respondent sample was skewed toward therapists and included only two Directors, which may bias the findings toward frontline clinical perspectives rather than strategic or organisational viewpoints. Additionally, participants classified as ‘Other’ included various roles, including Clinical Leads, Assistant Psychologists, Dementia Navigators, and Information and Education Advisors. The diversity of this group limits interpretation of the perspectives of individual professional groups, particularly those responsible for strategic or organisational decision-making. As some organisations contributed more than one respondent, the findings reflect respondents’ reports of service provision rather than independently verified service-level data.

### Implications for research and practice

Future research should prioritise under-represented regions of the UK, particularly Northern Ireland and Wales, to achieve more comprehensive UK-wide representation. Studies should also adopt targeted sampling approaches to capture a broader range of perspectives, particularly from service leaders and decision makers. Engaging a wider range of stakeholders, including commissioners, policymakers, and people living with dementia would help identify the structural, financial, and geographical barriers limiting access to consistent, evidence-based post-diagnostic support. Future work should explore how evidence-based interventions such as CST can be implemented and sustained more consistently across different services and settings, particularly in areas where post-diagnostic support provision remains limited. Additionally, future studies could develop and formally validate survey instruments to support ongoing national monitoring of post-diagnostic dementia services.

## Conclusion

This study identified variation in respondents’ reports of the availability and delivery of post-diagnostic support for people with dementia across the UK. Although evidence-based interventions such as CST are recommended in national guidance, respondents described barriers to their delivery, including workforce capacity, funding limitations and service commissioning arrangements. Strengthening post-diagnostic dementia care will require coordinated policy action, improved workforce support and clearer national standards to help ensure that people living with dementia can access timely, evidence-based support that promotes independence, helps manage symptoms and sustain quality of life following diagnosis.

## Supplementary Information

Below is the link to the electronic supplementary material.

Supplementary Material 1

Supplementary Material 2

## Data Availability

The datasets used and/or analysed during the current study are available from the corresponding author on reasonable request **.**.
