# Supplementary material for: Post-diagnostic support for dementia in the UK: mapping access, delivery and gaps in provision
Source: BMC Health Serv Res. 2026 Aug 8;26:1533. doi: 10.1186/s12913-026-15331-2 (PMC13737565; doi:10.1186/s12913-026-15331-2)
Supplement: Supplementary file 2 [file 12913_2026_15331_MOESM2_ESM.docx]

**Supplementary Material 1**

**Post-diagnostic Survey CRF**

Website for data-collection:

Qualtrics survey can be found here: <https://brainplus.qualtrics.com/jfe/form/SV_8BUTWwzLOzQWKaO>

Written text included in Qualtrics is as follows:

Which organisation do you work for?

________________________________________________________________

What is your role in your organisation?

- a.	Therapist (1)
- b.	Nurse (2)
- c.	Manager (3)
- d.	Director (4)
- e. Other (5)

**Display This Question:**

**If What is your role in your organisation? = e. Other**

What is your role in your organisation?

________________________________________________________________

Do you perceive the post-diagnostic support provided by your service for individuals diagnosed with dementia as satisfactory?

- Yes (1)
- No (2)

**Display This Question:**

**If Do you perceive the post-diagnostic support provided by your service for individuals diagnosed wi... = No**

Please state why.

________________________________________________________________

What types of post-diagnostic services does your service offer for patients diagnosed with dementia? (Please tick all those that are relevant)

- Care-coordination (1)
- Cognitive interventions	(2)
- Psychological support specific to dementia (3)
- Physical health services specific to dementia (4)
- Dementia information groups	(5)
- Social groups (walking/art/music groups, memory cafes etc) (6)
- Dementia information groups	(7)
- Other (8)

**Display This Question:**

**If What types of post-diagnostic services does your service offer for patients diagnosed with dement... = Other**

Please Specify:

________________________________________________________________

What is the primary focus of the post-diagnostic services provided

- Medical	(1)
- Psychological	(2)
- Social	(3)
- Other (4)

**Display This Question:**

**If What is the primary focus of the post-diagnostic services provided = Other**

Please Specify:

________________________________________________________________

Are these services available to patients immediately following diagnosis, or is there a waiting period?

- Immediately available (1)
- Waiting period (2)

**Display This Question:**

**If Are these services available to patients immediately following diagnosis, or is there a waiting p... = Waiting period**

Please state how long (in weeks).

________________________________________________________________

Do you offer any psychological support to people with dementia who are appearing anxious/depressed?

- Yes (1)
- No (2)

**Display This Question:**

**If Do you offer any psychological support to people with dementia who are appearing anxious/depressed? = Yes**

What support do you offer? (Please tick all that apply)

- Life story work	(1)
- Reminiscence/reality orientation	(2)
- Animal-assisted therapy	(3)
- Individual counselling/ psychotherapy	(4)
- Group psychotherapy	(5)
- Behavioural interventions	(6)
- Other (7)

**Display This Question:**

**If What support do you offer? (Please tick all that apply) = Other**

Please specify.

________________________________________________________________

How many psychologists do you have in your service?

________________________________________________________________

Is your service integrated with local dementia charities/community groups?

- No (1)
- Yes (2)

**Display This Question:**

**If Is your service integrated with local dementia charities/community groups? = Yes**

Please name the local dementia charities/community groups and their role.

________________________________________________________________

End of Block: Default Question Block

Start of Block: Group CST

Does your organisation deliver Cognitive Stimulation Therapy (CST)?

- No (1)
- Yes (2)

**Display This Question:**

**If Does your organisation deliver Cognitive Stimulation Therapy (CST)? = No**

Please select any reasons for why your organisation does not provide CST

- Insufficient funding (1)
- Have not heard of CST (2)
- Sceptical about its effectiveness (3)
- No available rooms to host (4)
- Staff shortages (5)
- Prioritization of other interventions/therapies (6)
- No patient demand (7)
- Other (8)
- I do not know (9)

**Display This Question:**

**If Please select any reasons for why your organisation does not provide CST = Other**

What other reasons does your organisation have for not providing CST? [please explain]

________________________________________________________________

**Display This Question:**

**If Please select any reasons for why your organisation does not provide CST = Prioritization of other interventions/therapies**

Please list any other interventions/therapies.

________________________________________________________________

**Display This Question:**

**If Does your organisation deliver Cognitive Stimulation Therapy (CST)? = Yes**

Do you personally deliver CST?

- No (1)
- Yes (2)

**Display This Question:**

**If Do you personally deliver CST? = Yes**

How often do you deliver CST?

- Never (1)
- Occasionally (2)
- Frequently (3)

**Display This Question:**

**If Do you personally deliver CST? = Yes**

How frequently do you deliver CST sessions per group (e.g. once a week)?

________________________________________________________________

**Display This Question:**

**If Do you personally deliver CST? = Yes**

How many CST groups do you run at a time?

- 1 (1)
- 2 (2)
- 3 (3)
- 4 (4)
- 5 (5)
- 6 (6)
- 7 (7)
- 8 (8)
- 9 (9)
- 10 (10)
- >10 (11)

**Display This Question:**

**If Do you personally deliver CST? = Yes**

What is the average size of a CST group?

- 1 (1)
- 2 (2)
- 3 (3)
- 4 (4)
- 5 (5)
- 6 (6)
- 7 (7)
- 8 (8)
- 9 (9)
- 10 (10)
- >10 (11)

**Display This Question:**

**If Do you personally deliver CST? = Yes**

Is there a waiting list for CST groups?

- No (1)
- Yes (2)

**Display This Question:**

**If Is there a waiting list for CST groups? = Yes**

Is there a waiting list for CST groups?

________________________________________________________________

**Display This Question:**

**If Do you personally deliver CST? = Yes**

How long do you spend preparing for CST sessions on average?

- Less than 15 minutes (1)
- 15 – 30 minutes (2)
- 30 – 60 minutes (3)
- 1-2 hours (4)
- More than 2 hours (5)

**Display This Question:**

**If Do you personally deliver CST? = Yes**

Use the scale to highlight your satisfaction.

|  | Extremely dissatisfied | Somewhat dissatisfied | Neither satisfied nor dissatisfied | Somewhat satisfied | Extremely satisfied |
| --- | --- | --- | --- | --- | --- |

|  | 0 | 10 | 20 | 30 | 40 | 50 | 60 | 70 | 80 | 90 | 100 |
| --- | --- | --- | --- | --- | --- | --- | --- | --- | --- | --- | --- |

| How satisfied are you with the current process of preparing CST sessions? () |  |
| --- | --- |
| How satisfied are you with the current methods of delivering CST sessions? () |  |

**Display This Question:**

**If Do you personally deliver CST? = Yes**

What factors influence the delivery of CST in your organisation?

- Number of patient referrals (1)
- Demand for support other than CST (2)
- Available funding (3)
- New research evidence (4)
- Other (5)

**Display This Question:**

**If What factors influence the delivery of CST in your organisation? = Other**

What other factors influence the delivery of CST in your organisation?

________________________________________________________________

**Display This Question:**

**If Do you personally deliver CST? = Yes**

What challenges or barriers do you have in implementing CST effectively?

________________________________________________________________

**Display This Question:**

**If Do you personally deliver CST? = Yes**

How do you address these challenges?

________________________________________________________________

End of Block: Group CST

Start of Block: Virtual CST

Is CST offered online at your service?

- No (1)
- Yes (2)

**Display This Question:**

**If Is CST offered online at your service? = Yes**

Do you personally deliver CST sessions online?

- No (1)
- Yes (2)

**Display This Question:**

**If Do you personally deliver CST sessions online? = Yes**

How many online CST groups do you run a year?

________________________________________________________________

End of Block: Virtual CST

Start of Block: Organizational

**Display This Question:**

**If Does your organisation deliver Cognitive Stimulation Therapy (CST)? = Yes**

**And What is your role in your organisation? = c. Manager**

**Or What is your role in your organisation? = c. Manager**

How many individuals trained in CST are employed in your organisation?

________________________________________________________________

**Display This Question:**

**If What is your role in your organisation? = c. Manager**

**Or What is your role in your organisation? = d. Director**

**And Does your organisation deliver Cognitive Stimulation Therapy (CST)? = Yes**

Is there a waiting list for CST groups?

- No (1)
- Yes (2)

**Display This Question:**

**If Is there a waiting list for CST groups? = Yes**

**And What is your role in your organisation? = c. Manager**

**Or What is your role in your organisation? = d. Director**

**And Does your organisation deliver Cognitive Stimulation Therapy (CST)? = Yes**

How long is the waiting list? (in weeks)

________________________________________________________________

**Display This Question:**

**If What is your role in your organisation? = c. Manager**

**Or What is your role in your organisation? = d. Director**

**And Does your organisation deliver Cognitive Stimulation Therapy (CST)? = Yes**

Where do you get funding for CST?

________________________________________________________________

**Display This Question:**

**If What is your role in your organisation? = c. Manager**

**Or What is your role in your organisation? = d. Director**

**And Does your organisation deliver Cognitive Stimulation Therapy (CST)? = Yes**

What factors influence your funding for CST? Please select all that apply.

- Competition for contracts (1)
- Government policy (2)
- Local funding opportunities (3)
- Level of demand for CST (4)
- Recent research evidence (5)
- Other (6)

**Display This Question:**

**If What factors influence your funding for CST? Please select all that apply. = Other**

Please specify.

________________________________________________________________

| Page Break |  |
| --- | --- |

**Display This Question:**

**If What is your role in your organisation? = c. Manager**

**Or What is your role in your organisation? = d. Director**

**And Does your organisation deliver Cognitive Stimulation Therapy (CST)? = Yes**

Are you expecting the funding for CST to change in the next year?

- No (1)
- Yes (2)

**Display This Question:**

**If Are you expecting the funding for CST to change in the next year? = Yes**

How are you expecting the funding situation for CST to change in the next year?

________________________________________________________________

| Page Break |  |
| --- | --- |

**Display This Question:**

**If What is your role in your organisation? = c. Manager**

**Or What is your role in your organisation? = d. Director**

**And Does your organisation deliver Cognitive Stimulation Therapy (CST)? = Yes**

Does your organisation ensure quality and consistency in delivery of CST across sessions and therapists? (e.g. Through patient attendance tracking, or session reports)

- Yes (1)
- No (2)

**Display This Question:**

**If Does your organisation ensure quality and consistency in delivery of CST across sessions and ther... = Yes**

How?

________________________________________________________________

**Display This Question:**

**If What is your role in your organisation? = c. Manager**

**Or What is your role in your organisation? = d. Director**

**And Does your organisation deliver Cognitive Stimulation Therapy (CST)? = Yes**

Are there any initiatives or strategies in place to expand access to CST?

- Yes (2)
- No (3)

**Display This Question:**

**If Are there any initiatives or strategies in place to expand access to CST? = Yes**

What are the initiatives/strategies in place to expand access to CST?

________________________________________________________________

**Display This Question:**

**If What is your role in your organisation? = c. Manager**

**Or What is your role in your organisation? = d. Director**

Does your organization collaborate with other entities (e.g., research institutions, healthcare providers, charities) to advance CST practices?

- Yes (1)
- No (2)

**Display This Question:**

**If Does your organization collaborate with other entities (e.g., research institutions, healthcare p... = Yes**

Who and how does your organisation collaborate with other entities (e.g., research institutions, healthcare providers, charities) to advance CST practices?

________________________________________________________________

End of Block: Organizational

Start of Block: Block 4

Thank you for taking part in our survey. If you would like your name to be put forward for a draw with an opportunity to win £25, please select yes, and you will be directed to another survey in which you can input your email address.  This is done so that we cannot link your responses to your email address.

- Yes (1)
- No (4)

**Display This Question:**

**If Thank you for taking part in our survey. If you would like your name to be put forward for a draw... = Yes**

Please click (or copy/paste the link in a new browser) to add your email address. https://brainplus.qualtrics.com/jfe/form/SV_dgSG2waVkOAHkNg
